# Skin and Soft Tissue Infections Caused by Methicillin-Resistant *Staphylococcus aureus* USA300 Clone

**DOI:** 10.3201/eid1308.061575

**Published:** 2007-08

**Authors:** Jennifer K. Johnson, Tina Khoie, Simone Shurland, Kristen Kreisel, O. Colin Stine, Mary-Claire Roghmann

**Affiliations:** * University of Maryland School of Medicine, Baltimore, Maryland, USA; † Veterans Affairs Maryland Health Care System, Baltimore, Maryland, USA

**Keywords:** Staphylococcus aureus, methicillin resistance, community-acquired infections, epidemiology, microbiology, staphylococcal skin infections, pulsed-field gel electrophoresis, research

## Abstract

Until recently, methicillin-resistant *Staphylococcus aureus* (MRSA) has caused predominantly healthcare-associated infections. We studied MRSA infections and overall skin and soft tissue infections (SSTIs) in outpatients receiving care at the Baltimore Veterans Affairs Medical Center Emergency Care Service during 2001–2005. We found an increase in MRSA infections, from 0.2 to 5.9 per 1,000 visits (p<0.01); most were community-associated SSTIs. Molecular typing showed that >80% of MRSA infections were caused by USA300. In addition, SSTI visits increased from 20 to 61 per 1,000 visits (p<0.01). The proportion of SSTI cultures that yielded MRSA increased from 4% to 42% (p<0.01), while the proportion that yielded methicillin-sensitive *S. aureus* remained the same (10% to 13%, p = 0.5). The increase in community-associated MRSA infections and the overall increase in SSTIs in our population suggest that USA300 is becoming more virulent and has a greater propensity to cause SSTIs.

## Abstract 2

An increase in SSTIs suggests that USA300 is becoming more virulent with a greater propensity to cause SSTIs.

Methicillin-resistant *Staphylococcus aureus* (MRSA) has been a cause of predominantly nosocomial or healthcare-associated infections. MRSA infections usually affect patients during hospitalization, after surgery, and during stays in long-term care facilities. In addition, MRSA infections are common in patients who have indwelling vascular catheters for dialysis or other medical treatments. However, during the past decade, multiple reports of community-associated MRSA (CA-MRSA) infections have been reported in patients who lack the above risk factors (*1*–*4*).

CA-MRSA infections differ from healthcare-associated MRSA (HA-MRSA) infections in a number of ways. CA-MRSA infections are predominantly skin and soft tissue infections (SSTIs), are often susceptible to other non–β-lactam antimicrobial drugs, and carry a type IV or V staphylococcal cassette chromosome (SCC) with the *mec*A gene. In contrast, HA-MRSA infections are found at multiple sites, are usually multidrug resistant, and carry the SCC*mec* types I, II, and III (*5*,*6*). In the United States, 2 major clones of CA-MRSA have been identified by pulsed-field gel electrophoresis (PFGE) and named USA300 and USA400 by the Centers for Disease Control and Prevention (CDC) (*7*). Among the community-associated types, the USA300 clone has recently emerged as the predominant cause of SSTIs in the United States (*8*,*9*). Toxin expression between CA-MRSA and HA-MRSA strains differs. Most CA-MRSA strains carry the intracellular toxin Panton-Valentine leukocidin (PVL), which is known for pore formation on polymorphonuclear cells of the host (*10*,*11*). In addition, the USA300 clone contains the arginine catabolic mobile element (ACME), which inhibits polymorphonuclear cell production (*10*).

In the summer of 2004, physicians in our Emergency Care Service (ECS) alerted us to an increased number of outpatients who had SSTIs caused by MRSA. This observation led us to begin this investigation with the following objectives: 1) to measure the incidence of MRSA infections in our ECS, 2) to describe these infections and their isolates, and 3) to measure the incidence of SSTIs in our ECS and the entire associated healthcare system over the past 5 years. We present molecular and epidemiologic evidence that the emergence of the USA300 clone has led to not only an increase in CA-MRSA infections but also an overall increase in SSTIs in our patient population.

## Methods

### Setting

The population for our retrospective study was derived from the Veterans Affairs Maryland Health Care System (VAMHCS), which provides comprehensive health care to >45,000 veterans in the Maryland area. Outpatient care is provided at 2 medical centers and 3 community-based outpatient clinics. Our ECS is located in the Baltimore VA Medical Center and serves ≈85 patients a day.

### MRSA Infections

We reviewed microbiology cultures obtained during an ECS visit from October 1, 2000, through September 30, 2005 (fiscal years [FYs] 2001–2005), in which MRSA was first isolated from a patient’s culture. The total number of ECS visits per FY was obtained from administrative records. The MRSA infection risk was calculated by dividing the total number of ECS patients whose culture results were MRSA positive for the first time by the number of ECS visits per FY. Information about patient demographics, clinical manifestations, and risk factors for nosocomial acquisition of MRSA was obtained from manual review of the VA’s computerized patient record system. Patients were categorized as to site of infection as follows: SSTI (wound culture positive for MRSA in the setting of new erythema, induration, warmth or pain at that site), urinary tract infection (positive urine culture in addition to at least 1 sign or symptom of a urinary tract infection), and pneumonia (positive sputum culture for MRSA in addition to a new infiltrate on chest radiograph). The infection was classified as healthcare-associated if the patient had a history of hospitalization, surgery, dialysis, or had been a resident in a long-term care facility within 1 year before infection or had a percutaneous medical device or permanent indwelling catheter at the time of infection. An infection in a patient without these risk factors was categorized as community-associated (*12*).

### MRSA Isolates

Clinical cultures were sent to the clinical microbiology laboratory of the VAMHCS. *S. aureus* was identified by following standard laboratory protocols. MRSA was defined as an *S. aureus* isolate that grew on oxacillin screen agar; methicillin-susceptible *S. aureus* (MSSA) was defined as an isolate that did not grow on oxacillin screen agar. Antimicrobial drug susceptibility was determined by following the methods and interpretation guidelines of the Clinical and Laboratory Standards Institute (*13*). Erythromycin-resistant and clindamycin-susceptible isolates were tested for inducible resistance by the D-test, following the guidelines that began on January 30, 2004. Clindamycin resistance data include all isolates that are truly resistant by breakpoint and isolates that have inducible resistance detected by the D-test. All isolates were frozen at –70°C in trypticase soy broth with 30% glycerol. MRSA isolates (n = 329) were typed by DNA sequencing analysis of the protein A (*spa*) gene hypervariable region as described (*14*). Allele identification was based on comparison with sequences in an *S. aureus* database (www.ridom.de/spaserver). PVL (*15*) and ACME (*10*) virulence factors were detected by following published protocols. PFGE was performed according to McDougal et al. (*7*). Photographic images of the gels were saved digitally with the Geldoc EQ (BioRad Laboratories, Hercules, CA, USA); gel analysis was saved with Fingerprinting II Software (BioRad Laboratories). The reference standard *S. aureus* NCTC 8325 was included in the first and fifteenth lane of each gel, and all isolates were normalized to this global standard. The band patterns were compared by means of the Dice coefficient by using the unweighted pair-group method to determine band similarity and following the criteria established by Tenover et al. to define the pulsed-field type clusters (*16*). We defined USA300 as isolates that had the MBQBLO repeat motif and were positive for PVL and ACME.

### SSTIs

For SSTIs, the total number of ECS visits (n = 3,688) and VAMHCS visits (n = 13,041) per FY, according to codes from the International Classification of Disease, Clinical Modification 9 (ICD-9-CM) (680, carbuncle and furuncle; 681, cellulitis and abscess of finger and toe; 682, other cellulitis and abscess; 704.8, folliculitis), were obtained from administrative records. The rate of SSTIs was calculated for the ECS and the VAMHCS by dividing the number of total visits for SSTIs by the number of visits per FY for each site. We also measured the number of patients who had SSTIs by taking the first patient visit for each year. Finally, we assessed whether patients’ infections were cultured during their visits for SSTI and whether that culture grew MRSA or MSSA.

### Statistical Analysis

Rates were computed as previously mentioned. Proportions were used to describe categorical variables and means to describe continuous variables. Categorical variables were compared by using χ^2^ or Fisher exact tests, as appropriate; continuous variables were compared by using Student *t* test. Statistical analysis was performed by using SPSS version 12.5 (SPSS Inc., Chicago, IL, USA).

## Results

**Figure 1:**
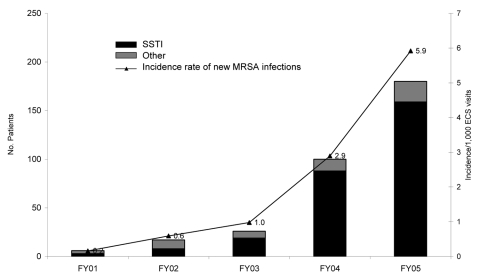
Methicillin-resistant *Staphylococcus aureus* (MRSA) infections in patients without a history of MRSA per 1,000 visits to the Baltimore Veterans Affairs Medical Center Emergency Care Service (ECS), 2001–2005. SSTI, skin and soft tissue infection; FY, fiscal year. FY01–03 versus FY04, χ^2^ test, p<0.001.

The proportion of ECS visits for MRSA infections for patients with no history of MRSA colonization or infection increased significantly from 0.2 per 1,000 ECS visits in FY01 to 5.9 per 1,000 visits in FY05 (p<0.01, χ^2^ test; Figure 1). The absolute number rose from 6 in FY01 to 180 in FY05, and 280 (81%) of 329 cases occurred during FY04 and FY05. In FY01, only 3 SSTIs were caused by MRSA compared with 159 in FY05.

The mean age of patients with new MRSA infections (n = 329) was 56 years; 98% were male, 69% were African American, 84% had an SSTI, 8% had a urinary tract infection, 2% had pneumonia, and 6% had other sites of infection. Of these 329 MRSA isolates, 76% were susceptible to clindamycin, 85% to tetracycline (n = 257), and 97% to trimethoprim-sulfamethoxazole. Overall, 217 (66%) of the 329 patients with MRSA infections had no known contact with the healthcare system in the year before their infection and most likely acquired MRSA in the community.

Molecular typing was performed on 296 (90%) of the 329 MRSA isolates from these infections. *Spa* typing showed a single dominant clonal type with the MBQBLO repeat motif (Table 1). The proportion of isolates tested with this *spa* type group significantly increased from 0% in FY01 to 89% in FY05 (p<0.01, χ^2^ test). Isolates that contained the virulence factors PVL and ACME also increased significantly from 0% in FY01 to 93% in FY04 and 89% in FY05 (p<0.01, χ^2^ test) and strongly correlated with isolates of the MBQBLO repeat motif. Molecular studies showed that isolates defined as USA300 by having the MBQBLO repeat motif and being positive for PVL and ACME increased from 0% in FY01 to 84% in FY05 and that USA300 was the dominant clone in FY03–FY05.

**Table 1:** Molecular typing of isolates from patients with new MRSA infections, Baltimore Veterans Affairs Medical Center Emergency Care Service, 2001–2005*

Time period	No. isolates tested	*spa* typing			PVL, no. (%)	ACME, no. (%)	MBQBLO motif, PVL, and ACME, no. (%)
		MBQBLO motif,† no. (%)	MDMGMK motif,‡ no. (%)	Other *spa* types,§ no. (%)			
FY01	2	0	2 (100)	0	0	0	0
FY02	13	4 (31)	5 (38)	4 (31)	3 (23)	3 (23)	2 (17)
FY03	21	15 (71)	6 (29)	0	15 (71)	15 (71)	15 (71)
FY04	94	77 (82)	14 (15)	3 (3)	74 (80)	70 (75)	68 (74)
FY05	166	147 (89)	15 (9)	4 (2)	154 (93)	147 (89)	138 (84)
Total	296	243 (82)	42 (14)	11 (4)	246 (83)	235 (79)	223 (76)

*MRSA, methicillin-resistant *Staphylococcus aureus;* PVL, Panton-Valentine leukocidin; ACME, arginine catabolic mobile element; FY, fiscal year. †*spa* types t008, t024, t112, t622, t064, t068, t121, t1881. ‡*spa* types t002, t045, t242, t548, t539. §*spa* types t018, t019, t084, t128, t160, t216, t937, t1887.

To confirm that isolates of the *spa* clonal type that had the MBQBLO repeat motif and were positive for ACME and PVL represent USA300, we performed PFGE on a subset of the isolates (n = 31). This subset consisted of a random selection of 10% of the total isolates. Sixteen isolates were positive for USA300 by both typing methods: 1) PFGE and 2) containing the MBQBLO repeat motif, PVL, and ACME. Three isolates had PFGE types similar to USA300 and PVL but had neither the MBQBLO repeat motif nor ACME. Twelve of the isolates did not have PFGE or *spa* types similar to USA300. No isolate was positive for MBQBLO repeat motif, PVL, and ACME and negative for USA300 by PFGE. When the MBQBLO that were ACME and PVL positive were compared with PFGE patterns for USA300, the sensitivity was 84% and the specificity was 100%; positive predicted value was 100% and negative predicted value was 80%.

**Figure 2:**
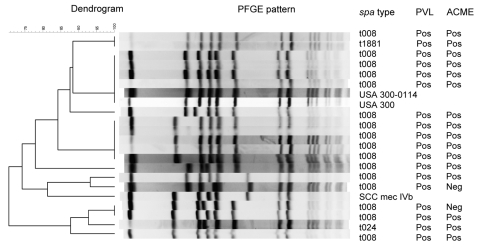
Pulsed-field gel electrophoresis (PFGE) of a stratified random sample of USA300 isolates and corresponding PCR results for Panton-Valentine leukocidin (PVL) and arginine catabolic mobile element (ACME). The Centers for Disease Control and Prevention’s PFGE results for USA300, USA300-0114, and SCC*mec* IVb were added as controls.

Not all isolates that were USA300 according to PFGE correlated with the MBQBLO repeat motif and were positive for PVL and ACME. One isolate had an unrelated *spa* type (BQBPO repeat motif), and 2 isolates were negative for ACME. Eighteen of these isolates had the MBQBLO repeat motif, and PFGE showed a similarity to PFGE type USA300 (Figure 2). PFGE in our study determined that all of these related USA300 isolates carried PVL and all except 2 carried the ACME virulence factor. The 2 without ACME were closely related to SCC*mec* IVb type. However, of the overall 329 MRSA isolates, 5 had the MBQBLO repeat motif and were PVL positive but ACME negative. Four isolates had the MBQBLO repeat motif (*spa* type t064) but were negative for PVL and ACME and similar to USA500 according to PFGE; these isolates were excluded from our definition of USA300.

**Figure 3:**
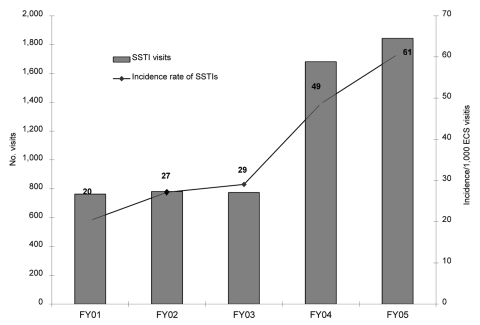
Visits for skin and soft tissue infections (SSTIs) in Baltimore Veterans Affairs Medical Center Emergency Care Service (ECS), 2001–2005. FY, fiscal year. FY01–03 versus FY04–05, χ^2^ test, p<0.001.

Because most of these MRSA infections were SSTIs and because our ECS physicians thought they were seeing more abscesses, we looked at the rate of SSTIs in the ECS. The rate of ECS visits for SSTI significantly increased from 20 per 1,000 ECS visits in FY01 to 61 per 1,000 visits in FY05 (Figure 3). Because of concerns that some of the same patients had multiple visits, we looked at patients with SSTIs in each year. The results were similar to SSTI visits and showed an increase over the years in the number of patients with SSTIs (Table 2). The absolute number and the proportion of patients for whom culture was performed increased from FY01–03 through FY04–05 (p<0001, χ^2^ test). We then examined the absolute number and the proportion of patients for whom cultures were performed and grew *S. aureus* (MRSA or MSSA). For our comparison of MRSA and MSSA, we chose this measurement to account for the increase in culturing. For MRSA, the absolute number and proportion of patients for whom cultures were performed and grew MRSA increased significantly from FY01–03 through FY04–05 (p<0001, χ^2^ test). For MSSA, the absolute number of patients for whom cultures were performed and grew MSSA increased from FY01–03 through FY04–05, but the proportion of patients for whom cultures were performed and grew MSSA remained the same (p = 0.10, χ^2^ test). Because of concerns that there may have been shifts in where care was delivered within our healthcare system, we also examined the number and rate of SSTIs for the entire VAMHCS. Absolute numbers of visits increased, with 2,020 visits in FY01, 1,972 in FY02, 2,190 in FY03, 3,337 in FY04, and 3,522 in FY05. The rate of SSTI visits also increased (2.75 SSTI visits per 1,000 visits in FY01–03 vs. 3.89 SSTI visits per 1,000 visits in FY04–05; p<0.001).

**Table 2:** Microbiologic characteristics of samples taken from patients with SSTIs, Baltimore Veterans Affairs Medical Center Emergency Care Service, 2001–2005*

Year	Had SSTI, no.	SSTI was cultured, no. (%)	SSTI was cultured and grew MRSA, no. (%)	SSTI was cultured and grew MSSA, no. (%)
FY01	496	98 (20)	4 (4)	10 (10)
FY02	574	120 (21)	10 (8)	12(10)
FY03	567	99 (17)	11 (11)	7 (7)
FY04	981	292 (30)	96 (33)	37 (13)
FY05	1,070	410 (38)	172 (42)	52 (13)

*SSTIs, skin and soft tissue infections; MRSA, methicillin-resistant *Staphylococcus aureus;* MSSA*,* methicillin-susceptible *S. aureus.*

## Discussion

During our 5-year study, we had an ≈4-fold increase in the incidence of MRSA infections, primarily SSTIs in people who had no risk factors for acquiring the infection from a healthcare setting. We showed that this increase was due to the USA300 clone and associated with an overall increase in SSTIs in our ECS and in the healthcare system as a whole. For these patients with SSTIs, the absolute number and proportion of those for whom a culture was performed and grew MRSA increased. We believe this reflects the increase in MRSA infections due to USA300. The absolute number of patients for whom a culture was performed and grew MSSA also increased, but the proportion remained the same. We believe the absolute numbers increased because more cultures were performed, not because MSSA infections increased.

Other reports of community-onset MRSA infections throughout the United States have been published (*5*,*8*,*17*–*19*). A recent publication by King et al. showed that USA300 was the predominant cause of SSTIs in the community (*8*). Carlton et al. also documented an increase in the number of total MRSA infections in San Francisco during 1996–2002 (*9*). This increase was shown to coincide with a statistically significant temporal increase in the number of community-onset MRSA infections. This study and our study support the hypothesis that CA-MRSA strains have factors that facilitate their spread in the community (*18*,*20*,*21*).

Despite the retrospective nature of our investigation, we were able to obtain 90% of the MRSA isolates for molecular typing. Molecular characterization of new cases of MRSA showed a dramatic increase in isolates with the MBQBLO repeat motif in the later years. This increase in these related *spa* types is consistent with the increase in the PFGE type USA300 seen by others (*6*,*19*). Not surprisingly, with the increase in the MBQBLO repeat motif, we also observed an increase in the virulence factors PVL and ACME. We did find 4 isolates of the *spa* type 65 that were negative for PVL and ACME and were similar to USA500 by PFGE and 5 isolates that were *spa* type 8 and positive for PVL but negative for ACME. This finding was not surprising because this phenomenon has been recently described; only isolates with the SCC *mec* IVa harbored the ACME gene (*22*).

We noted that SSTIs more than doubled during the 5 years of our investigation. The increase in SSTIs has also been observed throughout the United States. For example, a study by CDC showed that the visit rate for SSTIs during 2001–2003 was 410.7 per 10,000 persons (*23*). Although an overall increase in SSTIs was not seen, SSTIs in the ECS increased by 59% and for hospital outpatient department visits increased by 31%. These increases could be associated with the emergence of CA-MRSA infections and are consistent with our study findings, which showed that this increase was due to the USA300 clone and also with an overall increase in SSTIs.

The potential limitations of this study include the study population and its retrospective nature. Because the study population consisted of veterans who received treatment through the VA Maryland Healthcare System, and thus were mainly male patients of low socioeconomic status, the study was not a true population-based study. Although we focused only on the veteran population, we believe that our findings are consistent with those of other scientific studies and are relevant to most emergency department populations. The use of a veteran population is also an advantage. We were able to obtain more comprehensive medical information from the VA’s computerized medical record system than would likely be available for a nonveteran population. This study was a retrospective review of information obtained for the clinical treatment of infections, and therefore many SSTIs were not cultured. Although the increased frequency of culturing may have led to some increase in MRSA, the fact that the proportion of SSTIs that were MSSA stayed the same suggests that the increase in MRSA infections is real.

In conclusion, we showed an increase in CA-MRSA infections of the USA300 clone and an increase in SSTIs during a 5-year period in the ECS and systemwide at the VAMHCS. The emergence of the USA300 clone has led to not only to an increase in CA-MRSA infections but also an overall increase in SSTIs in our patient population. This finding suggests that this clone is becoming more virulent with a greater propensity to cause SSTIs.
